# Predicting the combined effect of multiple genetic variants

**DOI:** 10.1186/s40246-015-0040-4

**Published:** 2015-07-30

**Authors:** Mingming Liu, Layne T. Watson, Liqing Zhang

**Affiliations:** Department of Computer Science, Virginia Polytechnic Institute and State University, Blacksburg, VA USA; Department of Mathematics, Virginia Polytechnic Institute and State University, Blacksburg, VA USA; Department of Aerospace and Ocean Engineering, Virginia Polytechnic Institute and State University, Blacksburg, VA USA

## Abstract

**Background:**

Many genetic variants have been identified in the human genome. The functional effects of a single variant have been intensively studied. However, the joint effects of multiple variants in the same genes have been largely ignored due to their complexity or lack of data. This paper uses HMMvar, a hidden Markov model based approach, to investigate the combined effect of multiple variants from the 1000 Genomes Project. Two tumor suppressor genes, TP53 and phosphatase and tensin homolog (PTEN), are also studied for the joint effect of compensatory indel variants.

**Results:**

Results show that there are cases where the joint effect of having multiple variants in the same genes is significantly different from that of a single variant. The deleterious effect of a single indel variant can be alleviated by their compensatory indels in TP53 and PTEN. Compound mutations in two genes, *β*-MHC and MyBP-C, leading to severer cardiovascular disease compared to single mutations, are also validated.

**Conclusions:**

This paper extends the functionality of HMMvar, a tool for assigning a quantitative score to a variant, to measure not only the deleterious effect of a single variant but also the joint effect of multiple variants. HMMvar is the first tool that can predict the functional effects of both single and general multiple variations on proteins. The precomputed scores for multiple variants from the 1000 Genomes Project and the HMMvar package are available at https://bioinformatics.cs.vt.edu/zhanglab/HMMvar/

## Introduction

Identifying the deleterious effects of a variant is significant for disease studies. Different types of variation data have been identified with advances in sequencing technologies. Single nucleotide polymorphism (SNP) is the largest group of mutations in the variants identified so far in humans, and numerous methods have been developed for predicting the functional effects of SNPs. The second most common type of mutations is indel, referring to insertion or deletion of nucleotide bases. More and more indels have been discovered to be associated with diseases or cancers. Frameshift indels are expected to have large effects on protein functions (loss of function), since they change the reading frame of a gene thus change amino acids and probably the functions of proteins. Compared to SNPs, less work has been done on predicting the functional effect of indels.

Methods for predicting the functional effects of different types of variants are typically grouped into two classes [1], conservation-based predictor and trained classifier. Previous studies mainly concern SNPs, and a few dozen computer programs and web servers are devoted to predicting the effects of SNP variants. For example, SIFT SNP [2] is a conservation-based predictor and PolyPhen [3] is a trained classifier. Recent indel prediction studies include an evolutionary conservation-based approach for both coding and noncoding regions [4], a trained classifier method for frameshift variants [5], and another evolutionary conservation-based method for multiple types of variation [6]. A limitation of all these methods is that they only predict the effect of a single variant and cannot measure the functional effect of a set of variants in their entirety. Complex diseases are likely to be caused by multiple genes and/or multiple mutations on individual genes [7], so quantitatively measuring the effect of multiple variants together should be helpful for detecting causal genes/mutations for diseases. For example, it has been shown that the correlation between breast cancer and multiple SNPs of the ORAI1 gene is more significant than that with single SNPs [8]. The authors use a genetic algorithm to find combinations of SNPs along with their genotypes that are significantly different between the case group and the control group. The results reveal that new insights in cancer studies are possible by considering the joint effect of multiple variants or the associations among genetic variants. Other work [9, 10] concerns the variants C677T (alanine to valine) in the catalytic domain and A1298C (glutamate to alanine) in the regulatory domain of the methylenetetrahydrofolate reductase (MTHFR) gene, known to decrease the activity of the MTHFR gene and that patients could be inappropriately counseled for being at high risk for thrombotic episodes due to the difficulty of distinguishing between cis compound heterozygotes and trans compound heterozygotes. Therefore, it is important to study the joint effect of multiple variants.

This paper focuses on predicting the joint effect of variants from a single gene using a previously proposed hidden Markov model, HMMvar [11]. As the hidden Markov model is computed from the multiple protein sequence alignment for homologous proteins from different species, it reflects extent of evolutionary conservation naturally by its probabilistic profile. The probabilistic profile can be used to compute and compare the likelihood of generating mutant baring sequences given the HMM with the likelihood of generating mutant free sequences, i.e., wild type sequences, given the HMM. The lower the former compared with the latter, the more deleterious the mutants are likely. Therefore, HMMvar is able to predict the functional effect of a single mutation, as well as the joint effect of multiple mutations in coding regions.

To demonstrate the effectiveness of HMMvar, data from the 1000 Genomes project is used to identify genes that have multiple mutations, and HMMvar is used to predict the effect of multiple mutations on the genes identified. In addition, indels from two tumor suppressor genes, TP53 and phosphatase and tensin homolog (PTEN), are also used to investigate the effect of multiple indels from a single gene. If a frameshift indel occurs, it is possible that a nearby second indel rescues the gene by restoring the reading frame. There is a very limited knowledge about this kind of compensatory indels, but these are important because the deleterious effect of frameshift indels could be minimized by nearby compensatory indels. Hu and Pauline [5] claim that frameshift indels near each other are more likely to restore the translation frame. The present work found compensatory indel sets for TP53 and PTEN and measured the functional effects of individual indels and compensatory indel sets using HMMvar.

## Materials and methods

### Determine haplotypes by genotypes for the 1000 Genomes Project data

**Fig. 1:**
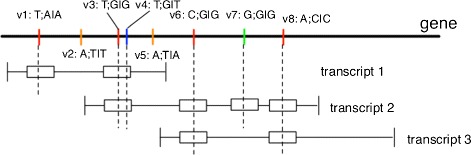
An example of variant classification in terms of genotypes. The *colored sticks* on the gene represent variants at different locations. *Colors* represent different classes of variants. The format *v*1:*T*;*A*|*A* means variant *v*1’s ancestral allele is *T* and the genotype is *A*|*A*, the same as other variants. The *boxes* on the transcripts represent exon regions. The gene and the transcripts share the same coordinate system

All the variants from the 1000 Genomes project Phase I along with their genotypes and ancestry alleles are collected to find the determined haplotype of an individual from a single gene. In order to quantitatively measure the effect of multiple variants on the same gene, the variant sets are formed in terms of their genotypes and the corresponding ancestral alleles. Given a certain gene and an individual sample, the variants are grouped into four classes based on the location and genotype. Figure 1 illustrates the classification of variants from a gene with three transcripts.

- Class 1: variants that are in the coding regions and the genotypes are homozygous and different from the ancestry allele, as the red variants shown in Fig. 1.
- Class 2: variants that are in the coding regions and the genotypes are homozygous and the same as the ancestry allele, as the green variants shown in Fig. 1.
- Class 3: variants that are in the coding regions and the genotypes are heterozygous, as the blue variants shown in Fig. 1.
- Class 4: variants that are not in the coding regions, such as 3’-utr, 5’-utr, or intron regions, as the orange variants shown in Fig. 1.

Only the variants in class 1 are kept as a set to be scored, because all the variants in Class 1 are homozygous and are mutants compared to the ancestral alleles. They can form a determined haplotype for a sample individual. As shown in the Fig. 1 example, a variant set is formed for each of the transcripts: Transcript 1 contains variant set {*v*_1_,*v*_3_}; Transcript 2 contains variant set {*v*_3_,*v*_6_,*v*_8_}; Transcript 3 contains variant set {*v*_6_,*v*_8_}. Finally, these sets will be scored against the corresponding transcripts by HMMvar. The homozygotes detected in individual samples along with the set score are available in the database for further analysis.

For each gene (transcript by considering alternative splicing), all homologous genotype variants that are different from the ancestry allele are identified based on an individual sample. As a result, a transcript related to a certain gene might be associated with multiple variant sets due to the difference of genotypes among samples, and a variant set can also be associated with multiple transcripts due to alternative splicing. Table 1 shows an example illustrating the relationship between individual, gene, and variant set. Only the records related to two individuals are shown here as an example (there are actually 2566 records related to gene ABCB5). As shown, gene ABCB5 is associated with multiple variant sets and even the same transcript (NM_178559.5) is associated with multiple variant sets due to the difference of genotypes of different individuals. The same variant set corresponds to multiple transcripts and multiple individuals. Finally, processing all genes that contain at least one variant set with size greater than one yielded 67,109 variant sets from 8021 genes (14,917 transcripts) involving 1092 individual samples.

**Table 1:** Variants sets related to gene ABCB5

Individual ID	Transcript ID	Set ID
NA20805	NM_001163941.1	7619
NA20805	NM_001163942.1	3062
NA20805	NM_001163993.2	3062
NA20805	NM_178559.5	7619
NA20806	NM_178559.5	2807
NA20806	NM_001163993.2	3062
NA20806	NM_001163942.1	3062
NA20806	NM_001163941.1	2807
...	...	...

### Compensatory indels in TP53 and PTEN

The indels from two tumor suppressor genes, TP53 and PTEN, are collected from two databases, International Agency for Research on Cancer (IARC) [12] and Catalogue of Somatic Mutations in Cancer (COSMIC) [13]. The 4736 variations (3565 for TP53 and 1171 for PTEN) include frameshift or in-frame insertions, deletions, and complexes (both insertion and deletion take place simultaneously in one location) in coding regions (Table 2).

**Table 2:** Data description

Type	Database	
	IARC (TP53)	COSMIC (PTEN)
Insertion (in-frame)	90	7
Insertion (frameshift)	419	116
Deletion (in-frame)	364	43
Deletion (frameshift)	1016	283
Complex (in-frame)	94	8
Complex (frameshift)	53	19
Total	2036	476

The effect of a deleterious mutation at the sequence level could be compensated for or alleviated by another mutation. For example, frameshift caused by a one base pair deletion could be recovered by a one base pair insertion nearby. A compensatory indel set is two or more indels that combine to preserve the open reading frame [14]. To simplify the search for compensatory indels, we restrict the consideration of compensatory indel sets, preserving the open reading frame, to those satisfying four conditions: (i) the number of nucleotides inserted or deleted per indel is less than five; (ii) the length of each indel is not divisible by three; (iii) the combined length of all indels is divisible by three; and (iv) all indels in the set occur within 20 base pairs. A single variant in a compensatory indel set is corrected (preserves the reading frame) by combining all other variants in the set. This paper considers the compensatory indel sets that satisfy the above four conditions for each of the TP53 variants and PTEN variants. Dynamic programming was used to find compensatory indel sets for single variants, which is similar to a subset sum problem [15], but with three different sums (−3, 0, and 3). To bound the computational effort, the maximum size of a compensatory indel set is bounded at 10, and the maximum number of compensatory indel sets for each valid length (sums −3, 0, and 3) is bounded at 20. The effects of compensatory indels are evaluated by comparing the HMMvar score of a single variant (as the mutant type) with the HMMvar score of a compensatory indel set (as the mutant type).

### HMMvar

According to the theory of natural selection, different regions of a functional sequence are subject to different selective pressures. Multiple sequence alignment reveals this by residual conservation in certain positions. Some positions are more conserved than others and less tolerant to mutations. HMMvar [11] embodied this theory by using profile hidden Markov models [16] to predict the effect of mutations. A profile HMM captures the characteristics of a multiple sequence alignment, from which quantitative conservation information (a probability) is obtained. Thus, a high probability of generation from the profile HMM for the wild type sequence and a low probability for the mutant sequence suggest that the mutation might be deleterious. HMMvar measures the fitness of a sequence against the profile HMM that represents a set of homologous proteins (ideally only orthologous proteins from different species). So, it is natural for HMMvar to score a mutant sequence with one or multiple variants. This property also enables scoring the joint effect of compensatory indels (defined later in the section). The odds ratio

(1) $$ O=\frac{P_{w}/(1-P_{w})}{P_{m}/(1-P_{m})}  $$

is used to score the effect of indel mutations, where *P*_*w*_ (*P*_*m*_) is the probability that the wild type (mutated type) protein sequence could have been generated by the profile HMM trained on a homologous protein sequence set, usually calculated by the Viterbi algorithm. The higher the *O* is, the more deleterious the mutation or mutations. In general, when *O*>1, the mutation or mutations might be deleterious, when *O*≈1, the mutation or mutations might be neutral, and when *O*<1, the mutation or mutations might be beneficial. For scoring the effects of SNP mutation or mutations [11], the odds ratio score is not as reliable as the bit score (computed by the HMMER3 package) difference log2(*P*_*w*_/*P*_*m*_) used here for SNPs.

## Results

### Scoring multiple SNPs

**Fig. 2:**
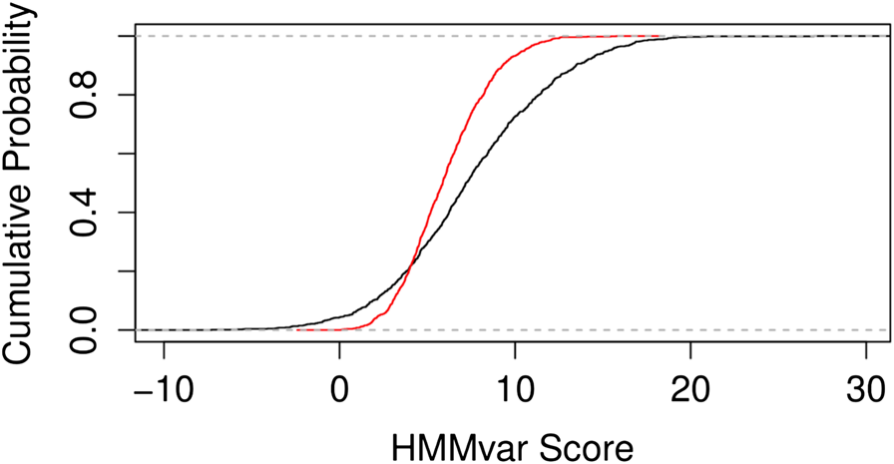
Comparison between variant set score (*black*) and single variant score (*red*)

Processing the variants from the 1000 Genomes project resulted in scoring 67,109 SNP sets. A SNP set may be formed from different transcripts, which results in multiple scores for a set (there are 91,970 set scores in total). For a SNP set and transcript pair, HMMvar measures the deleterious effect of the SNP set using the original transcript as the wild type sequence. 291,662 single variants from those SNP sets were gathered and scored. The mean set score distribution is significantly different from the single variant score distribution (one-tailed Wilcoxon rank-sum test, *p*<2.2×10^−16^). One thousand SNP set scores and 1000 single SNP scores are repeatedly sampled from 91,970 set scores and 275,840 single SNP scores. The cumulative distribution functions of the means of the set scores and single scores are shown in Fig. 2. The SNP sets are more likely to be scored higher than those of single SNPs. The density of SNP set scores tend to be higher than the density for individual SNP scores on both ends.

Let *V* = {*v*_1_,*v*_2_,…,*v*_*n*_} be a set of variants *v*_*i*_(1≤*i*≤*n*), *S* denote the HMMvar score of the set *V*, and *s*_1_, *s*_2_, …, *s*_*n*_ be the corresponding single variant scores of *v*_1_, *v*_2_, …, *v*_*n*_, respectively. Define *V* as a compensatory mutation (CM) set if *S*≤ min{*s*_1_,*s*_2_,…,*s*_*n*_}−1.5(max{*s*_1_,*s*_2_,…,*s*_*n*_}− min{*s*_1_,*s*_2_,…,*s*_*n*_}). One hundred eighteen CM sets were obtained from the data set. The CM sets indicate that the deleterious effect of a single variant is compensated by combining it with other variants.

Define *V* as a noncompensatory mutation (nonCM) set if *S*≥ max{*s*_1_,*s*_2_,…,*s*_*n*_}+1.5(max{*s*_1_,*s*_2_,…,*s*_*n*_}− min{*s*_1_,*s*_2_,…,*s*_*n*_}). Two thousand three hundred ninety-two nonCM sets were obtained from the data set. The nonCM sets indicate the joint effect of multiple neutral variants could possibly result in deleterious effect.

**Fig. 3:**
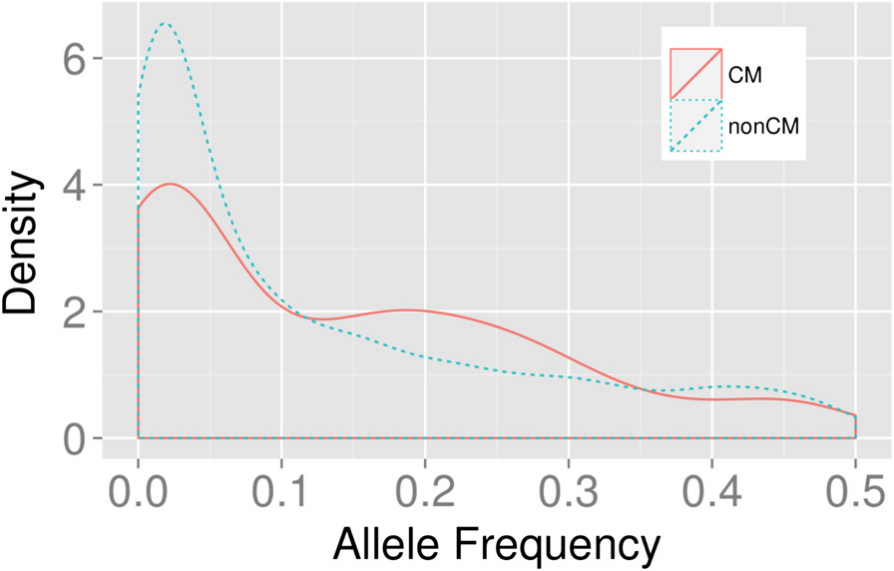
Allele frequency distribution of SNP variants in CM sets and nonCM sets

To investigate the single variants in the CM and nonCM sets, all the single variants from all the CM sets and all the nonCM sets are gathered, respectively. The allele frequency distributions from these two groups are compared in Fig. 3. When the allele frequency is less than 0.1, the proportion of the nonCM variants is greater than that of the CM variants. This is probably because the single variants are so deleterious that in most of cases, the joint effect of these deleterious variants is still deleterious. However, when the allele frequency is in the range of 0.1 to 0.3, the signal of the compensatory mutation effect is boosted.

As a test case for HMMvar’s capability in predicting the effect of multiple variants compared to the effect of single variants, the multiple mutations that have been shown to increase the severity of cardiovascular disease from single mutations are scored, in *β*-myosin heavy chain (MHC) and myosin-binding protein C (MyBP-C) genes. Studies have shown that single mutations in these two genes can lead to genetic cardiovascular disease, and multiple mutations on these same genes can lead to more severe cardiovascular disorders and even death [17]. As shown in Table 3 for both genes, compound mutations all have higher HMMvar scores than single mutations, consistent with the notion that compound mutations in these genes cause more severe cardiovascular disease than single mutations. The set score effectively reflects the cumulative effects of the single mutations. The maximum score for compound missense mutations in the *β*MHC gene is the combination of Arg719Trp and Met349Thr, which has been reported causing sudden death [17].

**Table 3:** Scoring multiple mutations in *β* MHC and MyBP-C genes

Gene	Mutation1	Score1	Mutation2	Score2	Set score
*β*MHC	Val39Met	1.7	Arg723Cys	3.4	5.0
*β*MHC	Pro211Leu	2.4	Arg663HIs	2.2	4.5
*β*MHC	Met349Thr	2.2	Arg719Trp	3.1	5.3
*β*MHC	Arg663His	2.2	Val763Met	2.3	4.4
*β*MHC	Arg719Gln	1.7	Thr1513Ser	0.0	1.6
*β*MHC	Asp906Gly	2.7	Leu908Val	2.0	4.6
MyBP-C	Gly5Arg	1.7	Arg502Trp	4.9	5.7
MyBP-C	Arg502Trp	3.9	Ser858Asn	2.4	6.4
MyBP-C	Alu542Gln	2.2	Ala851Val	2.2	4.4
MyBP-C	Asp745Aly	3.9	Pro873His	4.0	7.9
MyBP-C	Arg810His	2.9	Arg820Gln	2.6	5.5

### Scoring compensatory indels

**Fig. 4:**
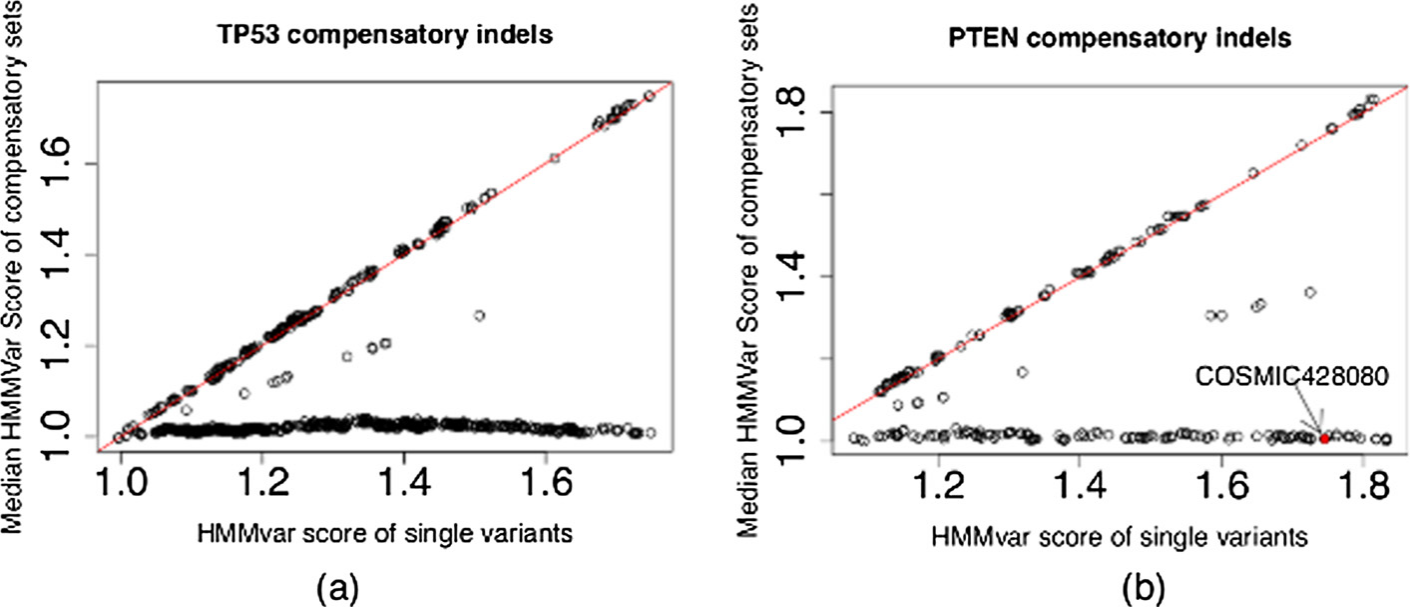
Scatter plot of HMMvar score of a single variant versus the median HMMvar score of the corresponding compensatory indel sets for the TP53 gene and the PTEN gene. The *red line* is *y*=*x*. **a** TP53 compensatory indels. **b** PTEN compensatory indels. The *red solid circle* marks the COSMIC variant with ID 428080

From TP53, 850 variants were found that met the criterion for belonging to a compensatory indel set, out of 3565 variants. The deleterious functional effects caused by these variants can be greatly weakened by compensatory indels as measured by HMMvar scores. There may be different compensatory indel sets for a given single variant due to different combinations. Figure 4a shows the HMMvar score of a single variant versus the median of the HMMvar scores of the corresponding compensatory indel sets. It is obvious that most of the deleterious variants (high HMMvar scores) are neutralized by the compensatory indel sets (low HMMvar scores ≈1).

PTEN is also an intensively studied tumor suppressor gene. Figure 4b shows the HMMvar score of 246 variants versus the median HMMvar score of the corresponding compensatory indel sets, which shows the same trend as the TP53 variants. This scoring procedure provides candidate compensatory indel sets, which when substituted for the indel, ameliorate the deleterious effect of that single mutation. For instance, the deleterious variant c.142delA (COSMIC428080) associated with skin cancer [18] has HMMvar score 1.75; however, with compensatory indels, the deleterious effect can be lessened to a HMMvar score of 1.07. At the same time, the results here demonstrate the importance of scoring multiple variants together, instead of individually, to understand their joint effect.

## Discussion

A single mutation, if not detrimental, can still exist in populations with low frequency. Over time, other mutations can also occur and thus multiple mutations can accumulate on the same gene. Compared to individual mutations, multiple mutations can be either more deleterious or less deleterious, the latter being known as compensatory mutation. Although it is not known which scenario is more prevalent in evolution, both scenarios have ample literature. Multiple mutations on the same gene, also called compound mutations, have been found to contribute or be linked to various genetic diseases (cf. [19–21]). In fact, a recent survey [17] of genetic cardiovascular disease led the authors to propose that multiple mutations, as opposed to single mutations, can be used as the genetic marker for the severity of cardiovascular disease, illustrating the importance of taking into account multiple mutations in disease outcome predictions. However, the current algorithms for predicting variant effect are limited to a single variant, a SNP or an indel. To fill this research gap, the present work proposes extending HMMvar, a hidden Markov model-based scoring method [11], to predict the effect of any number of mutations in any combinations (i.e., SNPs and/or indels).

**Fig. 5:**
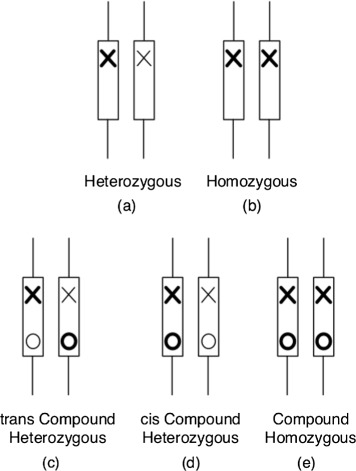
Zygosity of disease-causing single (**a**–**b**) or multiple mutations (**c**–**e**). The *cross* and *circle* indicate different mutations; the different *thicknesses* indicate different alleles of a mutation, and the *thicker* one indicates a disease-causing allele; the *boxes* represent a gene

Results show that multiple mutations do tend to have different effects on genes compared to single mutations, as reflected by the significant difference in the distributions of the HMMvar scores (one-tailed Wilcoxon rank-sum test, *p*<2.2×10^−16^). The HMMvar scores of multiple SNPs tend to be larger than those of single SNPs, suggesting that many of these multiple mutations exacerbate the deleterious effect of single mutations. Note that while scoring the SNPs discovered in the 1000 Genomes project, the scored variants are identified by the next-generation sequencing (NGS) data where short sequences are generated and compared to the human reference genome to identify variants. Therefore, the genotype of the mutant individual is unknown, as is whether multiple mutations exist on the same allele or different alleles. To circumvent this problem, only those variants that are in homozygous state are scored. Figure 5 shows the zygosity of disease-causing mutations or any mutations in general. Single variants could be in a heterozygous (a) or homozygous (b) state. For multiple variants on the same gene (Fig. 5c–e shows two mutations as an example), there are three possible scenarios: trans compound heterozygous (c), cis compound heterozygous (d), or compound homozygous (e). This study scored compound mutations as scenario (e), so the two mutations are linked on the same allele. Homozygous mutations, single or multiple, cannot be detrimental as individuals with them in homozygous state will not be able to survive.

When multiple mutations occur and accumulate on the same gene, it is possible that though deleterious by themselves, they come together and become less deleterious or even beneficial to the carrier due to either recovery of the original gene function or gain of new function. This type of mutation, known as compensatory mutation, has been documented in the literature with many of the cases found in bacteria and viruses [22–24]. Potential compensatory indels were identified in two tumor suppressor genes, TP53 and PTEN, where compensatory indels are composed of frameshift indels that can recover the original reading frame. Results show that the HMMvar scores for the effect of compensatory indels are indeed much lower than the scores of the frameshift indels, with many of them close to one (Fig. 4), suggesting that compensatory indels can rescue the deleterious effect of frameshift indels. Similarly, Fig. 3 shows that SNPs with putative compensatory effect (CM) tend to have higher frequencies in the 1000 Genomes data than those SNPs predicted to be noncompensatory (nonCM, Fig. 3).

HMMvar can predict the effect of a set of multiple variants in its entirety. This is especially useful when multiple variants occur in a protein, each of which may have deleterious effects on the protein function, but the combination of them may be less deleterious due to a compensatory effect. Profile HMMs, used as proposed, have the capability to predict the joint effect of multiple mutations along the gene given a specific haplotype. Due to current technological limitations, inferring genotypes of a gene is still a challenge and little data exists that can be used for understanding the effect of multiple variations on the same gene. With future sequencing technology, long sequences may be generated and genotypes of a gene may be determined with certainty, in which case the HMMvar method will be of great use in understanding the joint impact of multiple mutations, in addition to single mutations, and better identification of disease contributing/causing variations.
